# Estimating actual striking forces using attenuation properties of Taekwondo protectors

**DOI:** 10.1371/journal.pone.0328749

**Published:** 2026-01-13

**Authors:** Minho Chae, Jonghak Hwang, Woosup Han, Sihyun Ryu, Sangkyoon Park, Sukyung Park

**Affiliations:** 1 Department of Mechanical Engineering, Korea Advanced Institute of Science and Technology, Yuseong-gu, Daejeon, Republic of Korea; 2 Research Department of Sport Industry, Korea Institute of Sport Science, Songpa-gu, Seoul, Republic of Korea; 3 Technology Innovation Team, Korea Sports Promotion Foundation, Kiheung-gu, Yongin-si, Gyeonggi-do, Republic of Korea; 4 Motion Innovation Center, Korea National Sport University, Songpa-gu, Seoul, Republic of Korea; Ordu University, TÜRKIYE

## Abstract

**Background:** Protectors significantly attenuate impact forces, yet their quantitative characteristics remain poorly understood. While accurately measuring actual striking force is essential for performance evaluation, practical and safety challenges prevent direct measurement on athletes. The protector’s attenuation obscures the true impact, necessitating a method to estimate the original force from the attenuated data.

**Methods:** A controllable pendulum-based impact testing apparatus was developed to evaluate force attenuation characteristics of Taekwondo body protectors. The system delivered repeatable impacts across varying magnitudes and contact durations to a mannequin equipped with a protector. Simultaneous measurements of input and transmitted forces through the protector enabled quantification of attenuation ratios under controlled conditions. To demonstrate practical application, five Taekwondo athletes performed standardized kicks on the instrumented protector setup, allowing estimation of their actual striking forces using the derived attenuation factor.

**Results:** Force attenuation ratios demonstrated high consistency across impact magnitudes ranging from 300 to 5800 Newtons, with impact duration variations showing minimal influence on attenuation performance. Linear attenuation relationships were established between input and transmitted forces ($R^{2}\geq 0.987$), enabling derivation of a predictive attenuation factor. Validation testing with the pendulum system showed that the estimated input force trajectories achieved an nRMSE of 3.5%. Averaged across the entire force range, the MAPE was 4.7% for maximum force, 1.4% for impact duration, and 3.5% for impulse. Applying the attenuation factor to standardized kicks performed by five Taekwondo athletes yielded estimated maximum striking forces ranging from approximately 1300 to 1800 Newtons.

**Conclusion:** This study establishes the first quantitative characterization of impact attenuation in Taekwondo body protectors, providing a validated attenuation factor for estimating striking forces from transmitted forces. These findings demonstrate a practical methodology for quantifying striking forces that are otherwise difficult to measure.

## Introduction

In recent years, global interest and participation in combat sports have continued to rise [1]. In disciplines such as Taekwondo, precise measurement of an athlete’s striking force is indispensable for performance analysis, training optimization, injury prevention, and related objectives. To measure these forces while safeguarding athletes, the force sensors are routinely covered with a protector or cushioning material. However, since the protector absorbs and attenuates part of the applied force, considerable discrepancies arise between the actual striking force delivered by the athlete and the force measured by the sensors. This attenuation effect continues to pose significant challenges for accurately quantifying striking forces while wearing protective gear.

Previous studies have employed diverse systems [2] including water-filled heavy bag systems [3,4], force plate systems [5–7], strain gauge-based systems [8–10], accelerometer-based systems [11–13], load cell systems [14–16]. Nevertheless, large discrepancies—often exceeding an order of magnitude—have been reported between maximum force values depending on the measurement system used, highlighting ongoing limitations in measurement consistency and reliability. In many studies, the protective padding or damping material mounted on top of the force sensor differs in thickness and composition, causing the cushioning effect to vary across experimental protocols. This variability makes it difficult to measure striking forces accurately and consistently, or to compare results between studies. Alternatively, several studies have measured impact forces using methods that entirely eliminate the cushioning effect [17–19], most commonly by estimating impact forces from pre-impact velocity rather than by employing force transducers. However, such approaches, which estimate force from pre-impact velocity or acceleration, can only provide total impulse and fail to capture the force–time profile, such as maximum force or contact duration. They also neglect the complex multi-joint dynamics involved in actual striking movements, limiting their accuracy. Some of these studies attempted to directly measure impact forces by attaching flexible pressure sensors to the foot or striking body part, aiming to avoid injury risk [20,21]. However, such sensors typically estimate the total contact force based on localized pressure readings, which may not accurately represent the distributed force profile across the entire contact surface, especially under dynamic impact conditions. Conversely, several studies have employed striking machines to evaluate the damping effect of protective equipment such as boxing gloves; however, most of these works attached sensors to only one side of the impact apparatus (either the striker or the target) and inferred the forces and impulses acting on the opposite side indirectly [22–28]. To the best of our knowledge, no previous study has quantified the attenuation of force profiles through protectors by simultaneously mounting sensors on both the striking and impacted sides. The present work addresses this gap by instrumenting the inner (impacted) and outer (striking) surfaces of the protector with force transducers, thereby enabling accurate estimation of the true force profile and systematic quantification of the protector’s force attenuation characteristics.

In this study, we investigated how input striking forces are attenuated by Taekwondo protectors. To quantify impact magnitude and contact dynamics through force transmission, we developed a pendulum-based impact apparatus capable of replicating a range of striking scenarios with coupled variations in force magnitude and contact duration. By instrumenting force transducers on both the striking and impacted surfaces, we measured input and transmitted force profiles. From these data, we derived consistent attenuation factors that characterize the force attenuation properties of the protector. Finally, we applied these factors to transmitted force measurements obtained from actual Taekwondo athletes performing kicks, thereby enabling estimation of the original striking forces that would otherwise be difficult to measure directly.

## Materials and methods

The experimental procedure consisted of three main stages: (1) development of a pendulum-based impact apparatus, (2) derivation of attenuation factors through controlled impact testing, and (3) estimation of athletes’ striking forces based on kicking experiments.

### Impact system development

#### Impulse–momentum-based design principles.

The following impulse–momentum relationships were used to design and analyze the pendulum-based impact system. When the pendulum is assumed to be stationary before impact, the impulse *J* is defined as:

$$J=I\Delta \omega =I\omega^{+}-I\omega^{-}=I(\omega^{+}-\omega^{-})$$ (1)

where *I* is the pendulum’s moment of inertia, and $\omega^{+}$ and $\omega^{-}$ are the angular velocities immediately after and before impact, respectively.

$$e=-\omega^{+}/\omega^{-}$$ (2)

Using this definition, the impulse can be expressed as a function of the angular velocity at impact

$$J=-I(1+e)\omega^{-}$$ (3)

Hence, if the coefficient of restitution *e* is known and the impact angular velocity of the pendulum is measured, the impulse can be calculated. Assuming pivot friction and other energy losses of the pendulum are negligible, the work–energy principle allows the angular velocities to be determined from the initial drop angle $\theta_{i}$ and the rebound angle $\theta_{f}$

$$\frac{1}{2}I(\omega^{-}{)}^{2}=mgl(1-\cos\theta_{i})$$ (4-a)

$$\frac{1}{2}I(\omega^{+}{)}^{2}=mgl(1-\cos\theta_{f})$$ (4-b)

where *m* is the mass of the pendulum, *g* is the gravitational acceleration, and *l* is the effective length of the pendulum to the center of mass. From Eqs (1), (2), (4-a) and (4-b), the impulse can be written as

$$J=I(\omega^{+}-\omega^{-})=-\sqrt{2mglI}(\sqrt{1-\cos\theta_{i}}+\sqrt{1-\cos\theta_{f}})$$ (5-a)

$$J=I(\omega^{+}-\omega^{-})=-(e+1)\sqrt{2mglI(1-\cos\theta_{i})}$$ (5-b)

Based on this relationship, the pendulum’s mass, effective length, and initial drop angle were chosen to achieve the target impulse range in the design.

#### Impactor unit.

**Fig 1:**
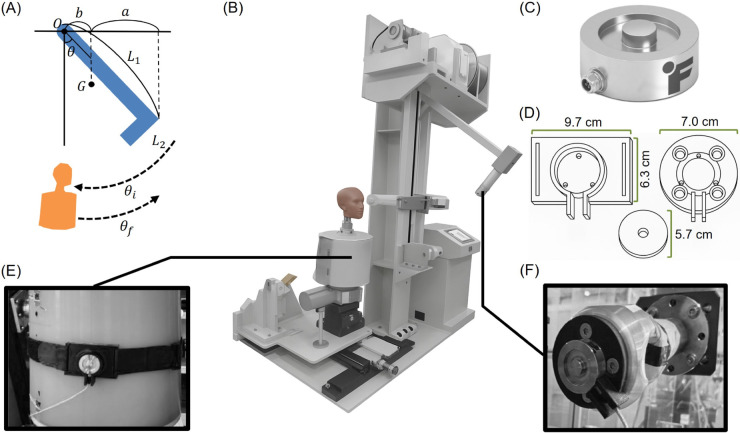
Experimental apparatus for measuring force attenuation in Taekwondo body protectors. (A) Design schematic of the pendulum-based impact apparatus, illustrating the swing trajectory and release angle (θ). (B) Overall view of the experimental setup, comprising a pendulum impactor, a mannequin outfitted with a Taekwondo body protector, and a force measurement system. (C) Load cell used for force measurement. (D) A custom-designed load cell adapter cap and mounting. (E) Transmitted force measurement using a load cell positioned behind the protector on the mannequin surface. (F) Input force measurement using a load cell mounted at the pendulum head.

To generate simple and repeatable impact forces, an impactor unit based on the free-fall pendulum principle was developed (Fig 1A, 1B). The system parameters were determined to achieve impulse values in the range of approximately 15–50 Ns, based on previously reported maximum forces and contact durations observed in human strikes (Table 1). Because impulse is governed by physical design parameters such as mass, moment of inertia, and release angle, the system enables stable and reproducible impact generation. The vertical pendulum was designed with adjustable mass of 10–15 kg, and a steel frame of 1.04 meters in length to ensure structural durability under repeated high-intensity impacts. The striking head, which contacts the target, was fabricated from aluminum, with a mass of 3.5 kg and a length of 0.385 meters (Fig 1B). The pendulum system allows fine manual adjustments of both horizontal and vertical alignment. A rotary encoder was integrated for real-time measurement of the release angle. The release and braking mechanism employs a powder clutch system to minimize structural vibration and to maintain consistent initial conditions throughout repeated trials.

**Table 1:** Summary of impact characteristics from previous and current studies on human striking actions. For each study, the type of measurement system and the corresponding striking action(s) are listed. When multiple actions were assessed within a study, they are separated by a tilde (~). Summary data are reported as mean (SD)

Study	Measurement	Action	Maximum Force (N)	Impact Duration (s)	Impulse (Ns)
Pieter and Pieter (1995) [3]	Water-filled heavy bag, built-in sensor	Taekwondo kicks and punch	341 (44) ~ 661 (52)	–	–
Girodet et al. (2005) [29]	Force sensor	Karate punch	1745	0.015	13.7
Pedzich et al. (2006) [30]	Force plate	Taekwondo kicks	7751 (2570) ~ 8569 (2381)	0.016 (0.002) ~ 0.018 (0.004)	60 (24) ~ 84 (30)
Gulledge & Dapena (2007) [31]	Force plate	Karate punches	790 (130) ~ 1450 (290)	0.022 (0.004) ~ 0.032 (0.008)	37.7 (14.6) ~ 43.2 (15.3)
Falco et al. (2009) [20]	Film sensor	Taekwondo kick	1304 (608) ~ 2089 (634)	–	–
Sullivan et al. (2009) [32]	Accelerometer	Taekwondo kick	5419 (659) ~ 6400 (898)	–	–
Estevan et al. (2011) [21]	Film sensor	Taekwondo kick	1203 (154) ~ 1829 (161)	0.022 (0.001) ~ 0.029 (0.002)	–
Neto et al. (2012) [14]	Load cell	Palm strike	957 (436) ~ 1884 (900)	–	–
Buśko et al. (2014) [9]	Dynamometer	Boxing punch	1634 (674) ~ 2899 (457)	–	–
Wasik & Nowak (2015) [18]	3D imaging	Taekwondo punch	1308 (173)	–	0.170 (0.007)
Svoboda et al. (2016) [33]	Dynamometer	Boxing punch	1970 ~ 2292	0.0109 ~ 0.0129	–
Buśko et al. (2016) [12]	Accelerometer	Boxing punch Taekwondo kick	848 (248) ~ 3514 (1190)	–	–
de Souza et al. (2017) [34]	Strain gauge	Karate punch	1812	–	–
Gavagan& Sayers (2017) [8]	Strain gauge	Taekwondo Karate Muay Thai kick	1211 (219) ~ 1547 (530)	–	30.9 (6.9) ~ 38.8 (15.3)
Lenetsky et al. (2018) [13]	Accelerometer	Punch	1411 (365) ~ 4749 (107)	–	26 (7) ~ 75 (8)
Beranek et al. (2020) [35]	Load cell	Punch Elbow strike Palm strike	2920 (1450) ~ 4120 (1660)	–	–
Jovanovski & Stappenbelt (2020) [15]	Load cell	Boxing punch	758 ~ 3845	–	–
Khasanshin & Aleksey (2021) [36]	Air Pressure	Boxing Punch	1006 (198)	–	–
Beranek et al. (2022) [37]	Force plate	Punches Elbow strike	3158 (1217) ~ 6460 (1612)	–	8.4 ~ 19.6
Finlay et al. (2023) [38]	Force plate	Boxing punch	1645 (537) ~ 2624 (581)	–	–
**Current study** **(estimated input)**	**Load cell**	**Taekwondo kick**	**1454.14 (258.96)**	**0.026 (0.002)**	**19.79 (4.51)**

#### Target unit.

The target unit consisted of a cylindrical aluminum mannequin (6061 alloy) designed to approximate the geometry of a human upper torso (Fig 1B). The mannequin was mounted on a pivot to allow free rotation, simulating the backward displacement of the human body upon receiving a kick. After each impact, an automated motorized return system rapidly reset the target to its initial position, ensuring both efficiency and consistency across repeated trials.

#### Measurement unit.

Impact forces were measured using an LLB 450 load cell (FUTEK, USA; maximum load: 22,241 N, natural frequency: 34 kHz, material: 17-4PH) (Fig 1C). A custom-designed adapter cap was installed on the load cell to ensure consistent contact with the protector’s curved inner surface and minimize misalignment. Preliminary tests using an instrumented hammer confirmed that the cap introduced no significant errors under moderate loading (Fig 1D). Prior to the main experiments, quasi-static and low-speed impact tests using an instrumented hammer confirmed that the adapter cap did not introduce significant measurement discrepancies under moderate loading conditions. Sensor signals were amplified using an ST-AM100 amplifier (Sensetech, UK) and recorded via a USB-6211 DAQ (National Instruments, USA). Data acquisition and control were performed using LabVIEW software. Input impact forces were measured using the load cell mounted on the pendulum head (Fig 1F), while transmitted forces were simultaneously measured by the load cell mounted behind the protector on the mannequin (Fig 1E). This dual-sensing configuration allowed quantification of the protector’s impact attenuation performance. Here, the ‘input force’ (also referred to as ‘striking force’) is defined as the actual force exerted by the striker onto the protector surface, and the ‘transmitted force’ as the attenuated force measured behind the protector. For clarity, ‘input force’ and ‘striking force’ are used interchangeably.

### Derivation of attenuation factors

To evaluate the impact attenuation characteristics of the body protector, repeated impact tests were conducted across a range of release angles, varying the pendulum’s initial angle from 20^∘^ to 80^∘^ in 10^∘^ increments. For each angle, five impact trials were performed in both increasing and decreasing angle sequences, resulting in five repetitions per condition. Force data collected from both the pendulum head and the load cell mounted behind the protector on the mannequin were sampled at 1000 Hz. To reduce drift and high-frequency noise, the signals were smoothed using a fourth-order Butterworth bandpass filter with a passband of 0.5–350 Hz.

Three metrics were defined to quantitatively assess the protector’s attenuation performance: Maximum Force, Impact Duration, and Impulse. Maximum Force was defined as the peak force recorded during each impact. Impact Duration was determined by identifying the time interval between the first and second instances when the force exceeded 2% of the maximum value. Impulse was calculated by integrating the force-time curve between these two time points.

To examine the linearity between input and transmitted forces, linear regression analyses were performed for each metric. To confirm linearity, the coefficient of determination (*R*^2^) was required to be 0.95 or greater. From the resulting linear relationships between the input forces (measured at the pendulum head) and transmitted forces (measured at the back of the protector), attenuation factors were derived for each impact parameter.

### Athlete kicking experiments

To estimate the actual striking forces of athletes based on the previously derived attenuation factors, additional kicking experiments were conducted. Five elite male Taekwondo athletes from Chungnam National University (height: 180.76 ± 4.27 cm, mass: 70.28 ± 3.56 kg, age: 20.6 ± 0.89 years) participated in the study. All participants were recruited on 29 May 2024 and were adult collegiate elite Taekwondo athletes. After receiving a full explanation of the study objectives and procedures, each participant provided written informed consent, and the study was approved by the KAIST Institutional Review Board (approval number: KH2024-089).

Following a 20-minute warm-up, each athlete performed 20 roundhouse kicks against a cylindrical sandbag that was fixed at its base. The roundhouse kick, a signature technique in Taekwondo [39], was selected for this study due to its relatively high kinematic consistency across individuals [40]. The impact forces generated by the kicks were measured using an LLB 400 load cell (FUTEK, USA; maximum load: 10,020 N, natural frequency: 30.7 kHz, material: 17-4PH), sampled at 1000 Hz. The height of the load cell was individually customized for each participant. Each athlete designated a target location on the sandbag that they perceived as optimal for delivering a kick, based on their individual height and leg length. The load cell and its mount (approximately 5 cm in diameter) were then securely attached at this athlete-specified location. The body electronic protector was subsequently fitted over the sandbag, covering the load cell assembly. No additional padding was placed over the mount; the safety of the setup was verbally confirmed with each athlete, who reported no significant pain before the experiment proceeded.

To ensure accurate targeting, the perimeter of the 5.7 cm diameter load cell mount was visually marked on the outer surface of the electronic protector. Before a trial, the athlete would state the intended striking area of their foot, and a 1 cm diameter ink spot was applied to this specific area. For a trial to be included in the final analysis, the entire 1 cm ink mark had to be located within the 5.7 cm diameter circle marked on the protector. Trials were also excluded if the athlete self-reported the kick as a mishit. Among the total 100 kicks performed, 43 trials (11, 10, 3, 13, and 6 trials per athlete, respectively) that met these criteria were included in the final analysis. Raw force data were analyzed without applying additional filtering to minimize the distortion of the force profile.

## Results

**Fig 2:**
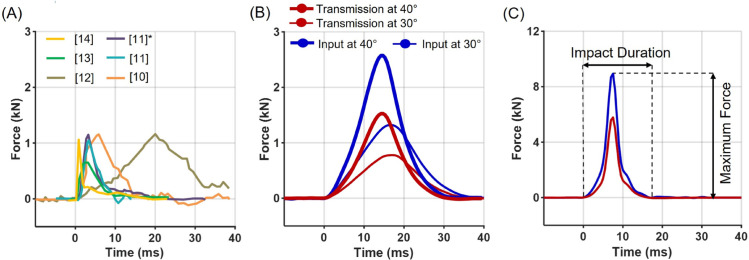
Profiles of impact force. (A) Reference force profiles of various strikes reported in previous studies, including Taekwondo kicks. (* Other strike types examined in the same study.) (B) Force profiles obtained from our pendulum impact apparatus at 30^∘^ and 40^∘^ release angles (thick solid lines). Input forces (blue) and transmitted forces through the body protector (red) are shown. (C) Force profiles from 80^∘^ free fall impacts, illustrating the definitions of maximum force and impact duration.

The impact-force profiles generated by the developed pendulum-based impact apparatus covered the range of force magnitudes and impact durations reported in previous studies on human Taekwondo kicks (Fig 2A, Table 1). By varying the pendulum’s free-fall drop height, our impact apparatus replicated 80–90% of the spectrum of impact forces (0.34–8.5 kN) and impact durations (10–32 ms) reported in previous studies. With increasing release angles, corresponding to higher collision velocities, maximum impact forces increased while impact durations decreased (Fig 2B, 2C). At lower angles, force profiles exhibited a triangular-like shape, whereas at higher angles, a short plateau was followed by a steep rise and rapid decay of the impact force.

**Fig 3:**
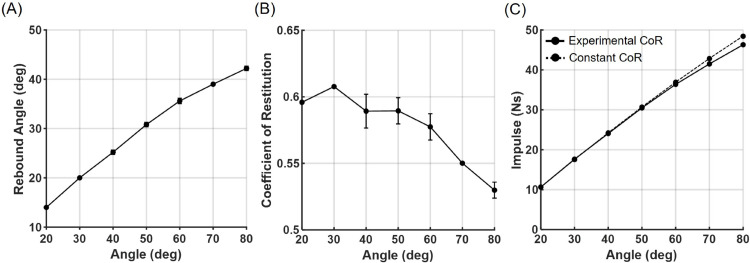
Performance evaluation of the impact apparatus. (A) Rebound angle of the pendulum after impact. (B) Experimentally calculated coefficient of restitution, e. (C) Corresponding estimation of impulse applied to the mannequin based on the pendulum motion. The dashed line in (C) indicates impulse estimation assuming a constant e = 0.6. Black dots represent the mean of five trials, and error bars indicate standard deviation

The impulse analysis of the pendulum-based impact apparatus demonstrated high consistency with the theoretical values used during system design (Fig 3). The pendulum lost energy during impact and rebounded to approximately 60% of its initial drop angle (Fig 3). The coefficient of restitution, e, calculated using the impulse-momentum relationship (Eq 1), showed a decreasing trend with increasing release angle (i.e., impact velocity) (Fig 3B). Although e is often assumed to be constant, the observed decrease at higher impact velocities likely reflects increased deformation and energy dissipation between the pendulum and the protector during high-speed collisions. The impulse exerted on the mannequin, calculated from the change in angular momentum of the pendulum, increased proportionally with impact velocity (Fig 3B). Compared to the impulse estimated under a constant e = 0.6 assumption, the experimentally observed reduction in e resulted in slightly higher impulse values (Fig 3C).

**Fig 4:**
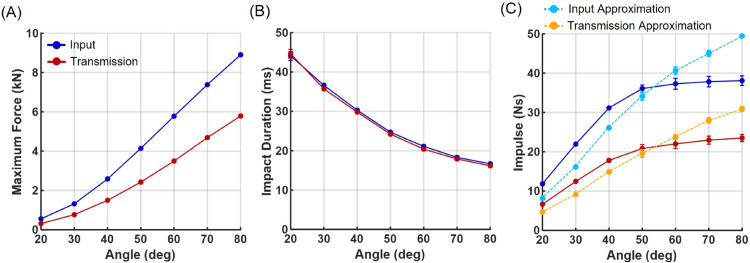
Input and transmitted impact-force metrics as a function of pendulum release angle. Experimental results of input (blue) and transmitted (red) forces. (A) Maximum impact force, (B) impact duration, and (C) impulse as a function of pendulum release angle. Approximated impulses, calculated as the product of maximum force and impact duration, are also shown for input (light blue) and transmitted (orange) forces. Each data point represents the mean of five repeated trials, with error bars indicating standard deviation. Overall, the very small error bars demonstrate the high repeatability of the test protocol.

Both input forces and transmitted forces through the protector exhibited similar trends with increasing pendulum release angle (Fig 4). In both cases, maximum impact force increased proportionally with release angle (i.e., impact velocity), while impact duration decreased accordingly. By varying drop heights, the developed system generated combinations of impact force and contact duration that encompass most of the impact conditions reported for Taekwondo kicks in previous studies.

However, with increasing impact velocity, the measured impulse values showed a tendency to plateau, which contrasted with the predicted impulse estimated from the difference in pendulum height before and after impact (Fig 3). Impulse is calculated as the integral of the force-time trajectory. When the force profile is approximated as a triangular waveform, impulse can be simply estimated as the product of maximum force and impact duration. This approximation yields a linear increase in impulse with release angle (impact velocity), which closely matches the impulse estimated from the pendulum’s rebound height (Fig 3). The observed plateau of measured impulse at higher velocities can be attributed to the steep force profiles observed in (Fig 2), where the brief duration of elevated force reduces the total integrated impulse. These results suggest that the impact forces measured by the load cell attached to the pendulum head may underestimate the true impulse during high-speed impacts. This discrepancy may arise from several experimental factors, including limitations in the dynamic response of the force transducer, signal filtering, and compliance in the sensor mounting structure. More detailed explanation of these factors is provided in the discussion section.

**Fig 5:**
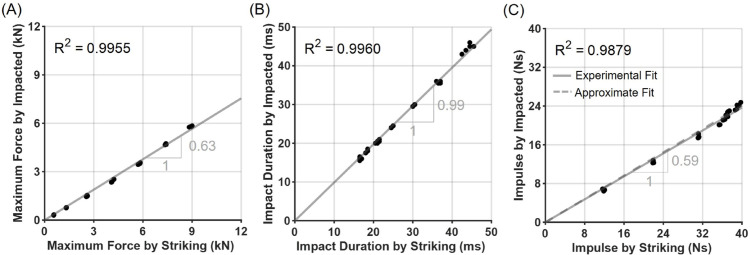
Relationships between input and transmitted impact parameters. The ratios between transmitted and input (A) maximum impact force, (B) impact duration, and (C) impulse are shown. Black dots represent individual test trials, and grey lines indicate linear regression fits.

Across a broad range of impact conditions, the transmitted forces measured through the body protector demonstrated highly consistent relationships with the corresponding input forces (Fig 5). Maximum impact force and impulse were attenuated by approximately 60% through the protector, whereas impact duration was almost identical between input and transmitted forces. The consistency and repeatability of these relationships enable the derivation of attenuation factors that can estimate the input force profile based on transmitted force measurements. The derived attenuation factors, the ratio of the transmitted force to the input force, were approximately 0.63 for maximum force, 0.99 for impact duration, and 0.59 for impulse.

**Fig 6:**
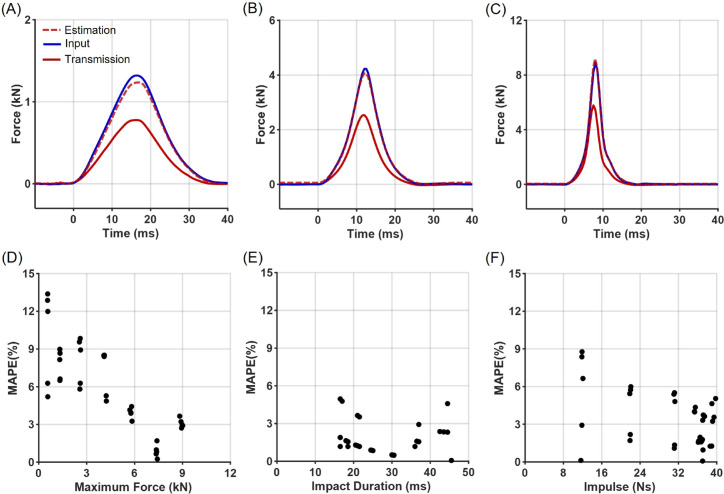
Validation of force estimation based on transmitted forces. (A-C) Examples of force profiles for input (blue), transmitted (red), and estimated input forces (red dashed) derived from transmitted forces using the attenuation factors. (D-F) Corresponding estimation errors for (D) maximum force, (E) impact duration, and (F) impulse, expressed as mean absolute percentage error (MAPE).

The estimated input forces, derived from the transmitted forces using the previously established attenuation factors, reproduced the input force profiles well (Fig 6A). The full force–time trajectory showed a mean nRMSE (normalized Root Mean Squared Error) of 3.5%, while the impact characteristics exhibited MAPE (Mean Absolute Percentage Error) within 15% for maximum force, 6% for impact duration, and 10% for impulse. Higher input force magnitudes were associated with improved estimation accuracy for maximum force, while no consistent trend was observed for impact duration and impulse. These estimation errors are primarily governed by the attenuation factors determined in Fig 5. For instance, applying a regression weighting that better reflects the 0–3 kN range, which corresponds to the typical impact forces reported for Taekwondo kicks, could enhance estimation accuracy within this lower force range; under this weighting, the mean maximum force error within the band falls to just 2.2%.

**Fig 7:**
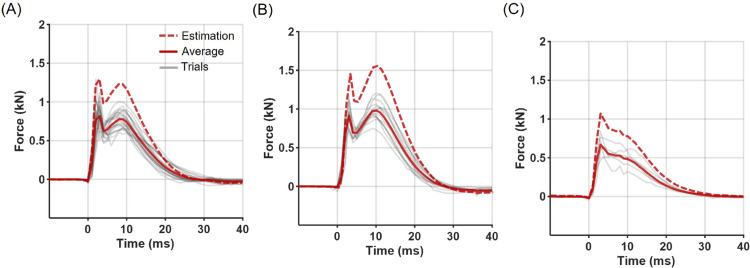
Measured and estimated Taekwondo kicks. The measured kick force profiles were categorized into (A) double-peaked profiles with a dominant first peak, (B) double-peaked profiles with a dominant second peak, and (C) single-peak profiles. Thin grey lines represent individual trials, red solid lines indicate the averaged transmitted force profiles, and red dashed lines show the estimated input force profiles reconstructed using the attenuation factors.

We applied the derived attenuation factors to athlete kick data to estimate the original striking forces (Fig 7). The kick force profiles were broadly categorized into three types: double-peaked profiles with a dominant first peak (Fig 7A), double-peaked profiles with a dominant second peak (Fig 7B), and single-peak profiles (Fig 7C). Double-peaked profiles accounted for the majority of kicks (84%), with the first peak being larger in 53% of cases and the second peak larger in 31% of cases. The maximum transmitted forces during kicks were approximately 0.916±0.16 kN, with corresponding impact durations of 25.48±2.33 ms and impulses of 11.73±2.68 Ns. These ranges correspond to the low-intensity region of the impact apparatus (release angle 20-30^∘^) for maximum force, the moderate-intensity region (release angle 40-50^∘^) for impact duration, and the low-intensity region (release angle 30^∘^) for impulse, respectively. Using the mean impact-force profile, the kick’s peak impact force was estimated to be approximately 1.4 kN, with a corresponding impact duration of approximately 26 ms and an impulse of approximately 20 Ns.

## Discussion

### Key findings

This study presents, to our knowledge, the first attempt to quantitatively evaluate the impact attenuation performance of Taekwondo body protectors using a free-fall pendulum system under repeatable dynamic impact conditions, and to extend this evaluation to estimate actual striking forces in athletes. The experiments demonstrated that both maximum force and impulse transmitted through the protector were attenuated by approximately 60%, while the impact duration remained largely unchanged. A highly linear relationship was consistently observed between input and transmitted forces across a broad range of impact magnitudes, allowing derivation of reliable attenuation factors that were successfully applied to estimate athletes’ striking forces.

### Interpretation of attenuation characteristics

The relatively consistent attenuation observed across a wide range of impact magnitudes suggests that the multilayer materials used in Taekwondo body protectors maintained stable energy absorption performance without exhibiting significant material nonlinearity under elevated impact loads. Typical protectors employ multilayer polymeric materials such as EVA foam and polyurethane foam, which are specifically designed to prevent rapid degradation of attenuation performance even after repeated impacts. These material properties likely contributed to the stable attenuation factors observed throughout the repeated impact tests in this study. Furthermore, while the cushioning materials effectively reduced maximum forces, they did not introduce significant dynamic deformation or rebound behavior within the short contact duration, which explains the minimal variation observed in impact duration across conditions.

Previous studies on boxing gloves have reported that repeated impacts lead to convergence of maximum force [41], impact duration, and impulse toward steady-state values. In consideration of this stabilization phenomenon, more than 500 pre-conditioning impacts were applied to the protector prior to attenuation factor measurements in this study.

Another important factor to consider is the fixed condition of the target unit used in the pendulum-based system. In general, when the impacted object remains stationary during collision, higher impact forces are recorded. In actual Taekwondo sparring, however, opponents tend to move backward reflexively upon receiving a strike; to replicate this behavior, the mannequin in the present setup was mounted on a release mechanism that allowed it to pivot and fall backward immediately after contact, thereby permitting additional attenuation through the motion of the target itself. Therefore, the attenuation factors derived in this study may provide a conservative estimate, potentially higher than the forces actually perceived by athletes during live competition.

Material properties and accumulated usage may also influence the attenuation factors depending on the manufacturer or wear history of the protector. While protectors have been introduced to promote objective scoring in Taekwondo, differences in scoring sensitivity and calibration protocols exist across manufacturers. Minimizing these inter-manufacturer discrepancies is desirable for fair competition, and the attenuation quantification methodology proposed in this study could serve as a foundation for establishing standardized performance evaluation criteria for body protectors.

### Interpretation of load cell measurement results

An adapter cap was mounted on the load cell to expand the sensing area and ensure consistent contact with the protector surface during impact measurements. This configuration demonstrated consistent performance during preliminary manual hammer tests under low-speed impact conditions. However, under high-speed impacts, discrepancies emerged between the impulses measured by the load cell and those estimated from the pendulum’s rebound angle, particularly at higher impact velocities where a plateau in impulse values was observed (Fig 4C). Considering that the bandwidth of the load cell sufficiently covers the frequency range of the impacts applied in this study, these discrepancies are more likely attributed to mechanical factors related to the adapter cap rather than limitations of the sensor’s electronic response. At high impact velocities, minor elastic deformation of the cap or transient micro-gaps between the cap and the load cell sensing surface may act as a mechanical filter, dispersing or attenuating the short-duration maximum forces. Such mechanical effects may have contributed to the observed plateauing of impulse measurements under high-speed conditions. Nonetheless, as both input and transmitted forces were measured using identical load cell configurations with identical adapter caps, the attenuation factors derived from the ratio between these forces are expected to be minimally affected by such artifacts. Therefore, the attenuation factors proposed in this study are considered applicable even under high-speed, high-intensity impact conditions.

The measurement system was designed to accommodate the structural constraints of embedding sensors inside and outside the protector while withstanding repeated high-intensity impacts. This imposed limitations on sensor selection and cap design. Although high repeatability was achieved, factors such as variations in impact angles or asymmetric strikes that may occur in actual competition settings were not fully addressed in this study.

### Validity and limitations of the pendulum-based impact system

In this study, a free-fall pendulum system was developed to quantitatively analyze the impact attenuation characteristics of Taekwondo body protectors. Compared to conventional impact simulation methods, the system offered substantial improvements in both safety and reproducibility, enabling systematic evaluation of the protector’s attenuation properties and allowing estimation of athletes’ actual striking forces. However, while this system was optimized for deriving attenuation factors, it has limitations in directly evaluating the sensing performance of the embedded electronic scoring systems used in Taekwondo competitions. Accurate evaluation of such scoring sensors requires test systems that more precisely replicate the dynamic characteristics of actual Taekwondo kicks, including not only a range of impact magnitudes but also controlled variations in impact velocity and contact duration.

This study focused on replicating the reported impact-force profiles observed during real Taekwondo kicks. Since the impulse generated by the pendulum is proportional to both its mass and velocity, the metal pendulum—being much heavier than a human foot—would generate excessively high forces if operated at velocities equivalent to real kicks [42]. This would pose substantial challenges to the durability of the target unit. Therefore, the pendulum velocity was intentionally limited to ensure that both maximum forces and impulse values remained within physiologically relevant ranges previously reported for Taekwondo strikes. For the purpose of evaluating scoring system performance, however, it would be necessary to replicate high-velocity, short-duration impacts similar to actual kicks. Achieving such conditions would require a motorized impactor system capable of independently controlling both force magnitude and contact duration, rather than relying on free-fall dynamics. Additionally, as suggested by the plateau phenomenon observed in the high-speed load cell measurements (Fig 4), further development of high-speed impact application and sensing systems is warranted for precise evaluation under such conditions.

### Interpretation of athlete striking force results

The actual striking forces estimated from the protector’s internal sensor measurements during athlete kicks were approximately 1.5 kN, which falls within the range of impact forces previously reported for Taekwondo kicks. This consistency supports the validity of the attenuation factor-based estimation approach proposed in this study. Unlike the artificial impact tests using mechanical devices, the athlete kicking trials exhibited a distinct double-peak pattern in the force profiles (Fig 7). Similar double-peak phenomena have been reported in previous studies involving impact apparatus with integrated cushioning materials [23,25,34]. Two primary mechanisms may explain this observation. First, stress wave reflections may occur when impact-induced stress waves propagate through the compliant backing structures (mannequin and protector) and are reflected back to the load cell with a time delay due to the system’s relatively low stiffness. Second, re-impact or rebound may arise as the elastic or viscoelastic deformation of the compliant mannequin and protector recovers following the initial impact, leading to secondary contact forces. An important consideration is whether the frequency range associated with these double-peak phenomena falls outside the relevant dynamic range of the attenuation factors derived in this study. Given that the cushioning materials generally exhibit limited dynamic frequency-dependent behavior, it is unlikely that these high-frequency components would meaningfully affect the validity of the attenuation factors. Therefore, the attenuation factors proposed here are considered applicable for estimating striking forces during actual athlete kicks despite the presence of double-peak patterns.

Furthermore, while it is impossible to quantify the accuracy of the estimated striking forces for athletes without a ground truth, the validity of the attenuation factor-based estimation model itself was validated through the controlled pendulum experiments. Additionally, only 43 of the 100 trials from the athlete kicking experiment were included in the final analysis. This was attributed to the difficulty participants had in precisely striking the 5.7 cm diameter load cell cap with a broad and variable contact area [43]. Such a low rate of effective strikes indicates that current precision measurement instruments are limited in their ability to assess the kind of unpredictable, real-time impacts characteristic of an actual competition.

### Conclusion

This study systematically quantified the impact attenuation performance of Taekwondo body protectors under repeated dynamic impact conditions and proposed a novel methodology for indirectly estimating athletes’ actual striking forces. The attenuation factor-based estimation approach developed in this study offers a new solution for quantifying striking forces that are otherwise difficult to measure directly, and may serve as a useful tool for performance assessment and training evaluation in Taekwondo athletes. Furthermore, the measurement and analysis framework established here may be applicable to evaluating the impact characteristics of other protective equipment with similar material compositions and structural designs. Future studies may also explore the development of motor-driven impact apparatus capable of replicating a broader range of impact conditions to further advance measurement capabilities.

## References

[1] Norjali Wazir MRW, Van Hiel M, Mostaert M, Deconinck FJA, Pion J, Lenoir M. Identification of elite performance characteristics in a small sample of taekwondo athletes. PLoS One. 2019;14(5):e0217358. doi: 10.1371/journal.pone.0217358 31150424 PMC6544235

[2] Beránek V, Votápek P, Stastny P. Force and velocity of impact during upper limb strikes in combat sports: a systematic review and meta-analysis. Sports Biomech. 2023;22(8):921–39. doi: 10.1080/14763141.2020.1778075 32677587

[3] Pieter PF, Pieter PW. Speed and force in selected taekwondo techniques. Biology of Sport. 1995;12(4):257–66.

[4] Fortin Y, Lamontagne M, Gadouas A. Punching bag dynamometer. In: Proceedings of the 14th International Society of Biomechanics Congress, Paris, France; 1993.

[5] Halperin I, Chapman DW, Martin DT, Lewthwaite R, Wulf G. Choices enhance punching performance of competitive kickboxers. Psychol Res. 2017;81(5):1051–8. doi: 10.1007/s00426-016-0790-1 27465395

[6] Loturco I, Nakamura FY, Artioli GG, Kobal R, Kitamura K, Cal Abad CC, et al. Strength and power qualities are highly associated with punching impact in elite amateur boxers. J Strength Cond Res. 2016;30(1):109–16. doi: 10.1519/JSC.0000000000001075 26110348

[7] Lapkova D, Pluhacek M, Adamek M. Numerical analysis of direct punch with a view to velocity and level of training. In: Proceedings of the International Conference on Numerical Analysis and Applied Mathematics (ICNAAM 2014). 2015. 550018-1–550018-4.

[8] Gavagan CJ, Sayers MGL. A biomechanical analysis of the roundhouse kicking technique of expert practitioners: a comparison between the martial arts disciplines of Muay Thai, Karate, and Taekwondo. PLoS One. 2017;12(8):e0182645. doi: 10.1371/journal.pone.0182645 28841670 PMC5571909

[9] Buśko K, Staniak Z, Łach P, Mazur-Różycka J, Radosław Michalski, Górski M. Comparison of two boxing training simulators. Biomedical Human Kinetics. 2014;6(1). doi: 10.2478/bhk-2014-0022

[10] Chadli S, Ababou N, Ababou A. A new instrument for punch analysis in boxing. Procedia Engineering. 2014;72:411–6. doi: 10.1016/j.proeng.2014.06.073

[11] Topal V, Ramazanoglu N, Yilmaz S, Camliguney AF, Kaya F. The effect of resistance training with elastic bands on strike force at Taekwondo. American International Journal of Contemporary Research. 2011;1(2):140–4.

[12] Buśko K, Staniak Z, Szark-Eckardt M, Nikolaidis PT, Mazur-Różycka J, Łach P, et al. Measuring the force of punches and kicks among combat sport athletes using a modified punching bag with an embedded accelerometer. Acta Bioeng Biomech. 2016;18(1):47–54. 27149957

[13] Lenetsky S, Brughelli M, Nates RJ, Cross MR, Lormier AV. Variability and reliability of punching impact kinetics in untrained participants and experienced boxers. J Strength Cond Res. 2018;32(7):1838–42. doi: 10.1519/JSC.0000000000002352 29420389

[14] Neto OP, Silva JH, Marzullo AC de M, Bolander RP, Bir CA. The effect of hand dominance on martial arts strikes. Hum Mov Sci. 2012;31(4):824–33. doi: 10.1016/j.humov.2011.07.016 22047701 PMC3274566

[15] Jovanovski A, Stappenbelt B. Measuring the boxing punch: development and calibration of a non-embedded in-glove piezo-resistive sensor. In: The 13th Conference of the International Sports Engineering Association. 2020. p. 13. 10.3390/proceedings2020049013

[16] Bolander RP, Neto OP, Bir CA. The effects of height and distance on the force production and acceleration in martial arts strikes. PMC3879635 24474886

[17] Walters S, Walters L, Hoffman B, Coltman CE, Mills DE. Validity and reliability of a novel impulse-based method to analyse human striking performance. J Sports Sci. 2025;43(9):842–51. doi: 10.1080/02640414.2025.2477855 40089841

[18] Wa¸sik J, Nowak K. Influence of different versions of the straight forward punch on the obtained force, energy and power – measurements of taekwon-do ITF athletes’ performance. 2015.

[19] Sørensen H, Zacho M, Simonsen EB, Dyhre-Poulsen P, Klausen K. Dynamics of the martial arts high front kick. J Sports Sci. 1996;14(6):483–95. doi: 10.1080/02640419608727735 8981287

[20] Falco C, Alvarez O, Castillo I, Estevan I, Martos J, Mugarra F, et al. Influence of the distance in a roundhouse kick’s execution time and impact force in Taekwondo. J Biomech. 2009;42(3):242–8. doi: 10.1016/j.jbiomech.2008.10.041 19124126

[21] Estevan I, Alvarez O, Falco C, Molina-García J, Castillo I. Impact force and time analysis influenced by execution distance in a roundhouse kick to the head in taekwondo. J Strength Cond Res. 2011;25(10):2851–6. doi: 10.1519/JSC.0b013e318207ef72 21912286

[22] Chadli S, Ababou N, Ababou A, Ouadahi N. Quantification of boxing gloves damping: method and apparatus. Measurement. 2018;129:504–17. doi: 10.1016/j.measurement.2018.07.036

[23] Lee B, McGill SM. Striking dynamics and kinetic properties of boxing and MMA gloves. Rev artes marciales asiát. 2014;9(2):106. doi: 10.18002/rama.v9i2.1175

[24] Beranek V, Stastny P, Bacik B, Bonkowski T, Novacek V. The effect of protective mat thickness on the upper limb strike force simulation in combat sports and self defense. J Hum Kinet. 2024;94:47–63. doi: 10.5114/jhk/192131 39563756 PMC11571458

[25] Perkins P, Jamieson A, Spratford W, Hahn A. Evaluation of ability of two different pneumatic boxing gloves to reduce delivered impact forces and improve safety. WJET. 2018;06(02):457–91. doi: 10.4236/wjet.2018.62028

[26] Girodet P, Durand F, Vaslin P, Lacouture P. Damping characteristics of nine samples of French boxing gloves. Computer Methods in Biomechanics and Biomedical Engineering. 2009;12(sup1):127–8. doi: 10.1080/10255840903080885

[27] Joseph R. A study of using boxing gloves with influence-damping properties. 2019;6(3).

[28] Therrien RG, Dessureault J. Deterioration with use of boxing gloves absorption characteristics. 2023.

[29] Girodet P, Vaslin P, Dabonneville M, Lacouture P. Two-dimensional kinematic and dynamic analysis of a karate straight punch. Computer Methods in Biomechanics and Biomedical Engineering. 2005;8(sup1):117–8. doi: 10.1080/10255840512331388533

[30] Dzich WP, Mastalerz A, Urbanik C. The comparison of the dynamics of selected leg strokes in Taekwondo WTF.

[31] Gulledge JK, Dapena J. A comparison of the reverse and power punches in oriental martial arts. J Sports Sci. 2008;26(2):189–96. doi: 10.1080/02640410701429816 17943591

[32] O’Sullivan D, Chung C, Lee K, Kim E, Kang S, Shin I. Measurement and comparison of taekwondo and yongmudo turning kick impact force for two target heights. PMC3879643 24474880

[33] Svoboda M, Soukup J, Jelen K, Kubový P. Measurement of force impact Taekwondo athletes, assessing the possibility of injury of human head. Procedia Engineering. 2016;136:211–5. doi: 10.1016/j.proeng.2016.01.199

[34] Souza VA d, Marques AM. Impact force of a reverse punch performed by karate athletes. Journal of Sports Science. 2023;1(1):1–10.

[35] Beranek V, Stastny P, Novacek V, Votapek P, Formanek J. Upper limb strikes reactive forces in mix martial art athletes during ground and pound tactics. Int J Environ Res Public Health. 2020;17(21):7782. doi: 10.3390/ijerph17217782 33114304 PMC7660618

[36] Khasanshin I, Osipov A. Using an artificial neural network to develop an optimal model of straight punch in boxing and training in punch techniques based on this model and real-time feedback. PLoS One. 2021;16(11):e0259457. doi: 10.1371/journal.pone.0259457 34843506 PMC8629283

[37] Beranek V, Stastny P, Novacek V, Słomka KJ, Cleather D. Performance level and strike type during ground and pound determine impact characteristics and net force variability. Sports (Basel). 2022;10(12):205. doi: 10.3390/sports10120205 36548502 PMC9785771

[38] Finlay MJ, Page RM, Greig M, Bridge CA. Test-retest reliability and sensitivity of senior elite amateur boxers maximal punch force, as quantified by a vertically mounted force plate. PLoS One. 2023;18(8):e0289791. doi: 10.1371/journal.pone.0289791 37561692 PMC10414587

[39] Moreira PVS, Falco C, Menegaldo LL, Goethel MF, de Paula LV, Gonçalves M. Are isokinetic leg torques and kick velocity reliable predictors of competitive level in taekwondo athletes?. PLoS One. 2021;16(6):e0235582. doi: 10.1371/journal.pone.0235582 34106936 PMC8189470

[40] Vagner M, Cleather DJ, Olah V, Vacek J, Stastny P. A systematic review of dynamic forces and kinematic indicators of front and roundhouse kicks across varied conditions and participant experience. Sports (Basel). 2023;11(8):141. doi: 10.3390/sports11080141 37624121 PMC10459763

[41] Perkins P, Jamieson A, Spratford W, Hahn A. Pneumatic boxing glove reduces upward drift in peak force and loading rate over a long series of impacts. WJET. 2019;07(01):18–53. doi: 10.4236/wjet.2019.71002

[42] Merk A, Resnick A. Physics of martial arts: incorporation of angular momentum to model body motion and strikes. PLoS One. 2021;16(8):e0255670. doi: 10.1371/journal.pone.0255670 34375352 PMC8354461

[43] Beranek V, Stastny P, Turquier F, Novacek V, Votapek P. Analysis of the contact area for three types of upper limb strikes. J Funct Morphol Kinesiol. 2022;7(2):50. doi: 10.3390/jfmk7020050 35736021 PMC9224799

